# *Leishmania infantum* Infection in Blood Donors, Northeastern Brazil

**DOI:** 10.3201/eid2204.150065

**Published:** 2016-04

**Authors:** Daniela C.S. Monteiro, Anastácio Q. Sousa, Danielle M. Lima, Raissa M. Fontes, Claudênia C. Praciano, Mércia S. Frutuoso, Loraine C. Matos, Maria J. Teixeira, Richard D. Pearson, Margarida M.L. Pompeu

**Affiliations:** School of Medicine of the Federal University of Ceará, Fortaleza, Brazil (D.C.S. Monteiro, A.Q. Sousa, R.M. Fontes, C.C. Praciano, M.S. Frutuoso, L.C. Matos, M.J. Teixeira, M.M.L. Pompeu);; School of Medicine of the University of Fortaleza, Fortaleza (D.M. Lima);; Unichristus School of Medicine, Fortaleza (M.S. Frutuoso);; University of Virginia School of Medicine, Charlottesville, Virginia, USA (R.D. Pearson)

**Keywords:** Leishmaniasis, blood donors, Leishmania infantum, parasites, Brazil, sand fly

**To the Editor:** *Leishmania infantum* is endemic to northeastern Brazil. It is responsible for visceral leishmaniasis (VL), a major emerging health problem in urban areas. Transmission occurs predominantly by the *Lutzomyia longipalpis* sand fly, but transfusion-associated VL, caused by *L. infantum*, has been reported from southern Europe and, by *L. donovani*, on the Indian subcontinent (*1*,*2*). Most *L. infantum* infections are asymptomatic (*3*), raising concern that the parasite could be present in donated blood from otherwise healthy residents in areas to which it is endemic (*4*).

VL caused by *L. infantum* is endemic to 20 of Brazil’s 27 states; an annual average of 3,553 cases occur nationwide, with 54% of all cases reported from Brazil’s northeastern region. The state of Ceará historically ranks first or second in number of cases; an annual average of 467 cases were reported during the last decade (*5*). Thirty-eight percent of cases were reported from Fortaleza, the capital, where 28.4% of the state’s population resides. Over a 10-year-period, 277 (7.8%) persons with VL have died, and 109 (39%) of VL-related deaths have occurred in Fortaleza. 

Sixty-nine percent of blood donors for Ceará reside in Fortaleza. To determine the prevalence of *Leishmania* infection among healthy blood donors, we tested blood donated to the State of Ceará Public Blood Bank. Compulsory serologic testing was also done for *Trypanosoma cruzi* (Chagas disease), hepatitis B and C, *Treponema pallidum* (syphilis), human T-cell lymphotropic virus types 1 and 2, and HIV-1 and -2. In the blood bank, 60% of units are centrifuged to separate the buffy coat in preparation for platelet separation. During May–November 2011, we randomly selected 431 buffy coats and tested them for *Leishmania* spp. by ELISA and PCR. To separate plasma from cells, 10 mL of buffy coat was centrifuged in Ficoll-Hypaque (Histopaque −1077, Sigma-Aldrich, São Paulo, Brazil). We tested plasma for leishmanial IgG by ELISA using a modified protocol of Evans et al. (*6*). IOC/L2906 *L. infantum* strain (MHOM/BR/2002/LPC-RPV) was used as the source of promastigote antigens. In addition, DNA was extracted from the mononuclear cell preparation, and PCR was performed with primers 150 (5′-GGG[G/T]AGGGGCGTTCT[G/C]CGAA-3′) and 152 (5′-[G/C][G/C][G/C][A/T]CTAT[A/T]TTACACCAACCCC-3′) that target the 120-bp conserved region of the *Leishmania* kDNA minicircle present in all *Leishmania* spp (*7*). As a positive control, kDNA was extracted from *L. amazonensis* promastigotes, strain BA-125 (MHOM/BR/87), characterized by PCR and isoenzymes (*8*). All PCR-positive samples were purified and sent to Ludwig Biotec (Alvorada, Brazil) for sequencing by ACTGENE-Molecular Analysis. The Federal University of Ceará Ethics Committee approved this study.

Buffy coats from 57 (13.2%) serum samples from 431 donors were positive for leishmanial IgG, and 20 (4.6%) were positive for *Leishmania* spp. DNA. Sequencing of all PCR-positive samples confirmed the *Leishmania* genus. Three donors tested positive by both ELISA and PCR. Overall, the prevalence of leishmanial infection was 17.1% of blood donors. Eighty of the 431 units tested positive for >1 of compulsorily screened infections and were rejected. Of the remaining 351 that were negative for co-infection, 43 (12.2%) were positive for leishmanial IgG and 15 (4.3%) for *Leishmania* spp. DNA. Two donors were positive for both by ELISA and PCR. The prevalence of *Leishmania* infection among blood units accepted for transfusion was 16%.

The results demonstrate a surprisingly high prevalence of *Leishmania* infection in blood donors in Fortaleza, several times higher than that other diseases for which blood is screened (Figure). In a recent study in Salvador, Brazil (*9*), 5.4% of blood donors had leishmanial antibodies, of which 68% were positive by its PCR targeting kDNA amplification.

**Figure:**
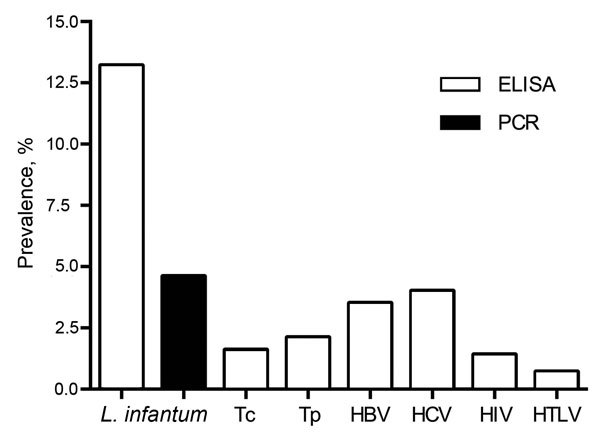
Comparison of the prevalence of *Leishmania infantum* as tested by PCR and ELISA and of other infections compulsorily tested in 431 blood donors in Fortaleza, state of Ceará, northeastern Brazil. HBV, hepatitis B virus; HCV, hepatitis C virus; HTLV, human T-cell lymphotropic virus; Tc, *Trypanosoma cruzi*; Tp, *Treponema pallidum*.

The percentages of antibody- or PCR-positive units capable of transmitting *Leishmania* and the outcomes are unknown. Viable *Leishmania* might not be in the blood of all PCR-positive donors, and even when present, the inoculum might be reduced by removal of infected circulating mononuclear phagocytes in the buffy coat, or parasites might be affected by steps involved in preparation or storage. However, if we consider units that test positive by PCR as being potentially infectious, the number of recipients at risk is of substantial concern. For example, in 2011, there were 99,933 blood donations to the State of Ceará Public Blood Bank. After compulsory screening for the other bloodborne pathogens, 93,238 units were accepted for transfusion. Extrapolating from the PCR-positive rate of 4.3%, a total of 4,009 recipients possibly were exposed to infection. Further studies are needed to determine whether recipients of blood from donors who are PCR positive and/or leishmanial antibody positive become infected with *L. infantum*. Persons with advanced AIDS or other immunosuppressive conditions seemingly would be at greatest risk for VL.

In Brazil, legislation requires that all blood for transfusion be tested for *T. cruzi* ., hepatitis B and C, *T. pallidum*, human T-cell lymphotropic virus types 1 and 2, and HIV-1 and -2. As additional information becomes available, screening for *L. infantum* also might be advisable to reduce the possibility of the recipient becoming infected, developing VL, and possibly being a reservoir of infection in the community (*10*), particularly in Ceará and other regions where the prevalence of *L. infantum* infection is high.
